# Other Lessons of Sickness

**Published:** 1889-08-31

**Authors:** 


### WORDS OF CONSOLATION.

### OTHER LESSONS OF SICKNESS.

(*For Reading to the Sick.*)

Besides giving us time to make our peace with God and prepare for eternity, which is the first and greatest use of sickness, it is also an opportunity for reflection on the way in which we perform the duties of everyday life. In some cases an illness comes as a positive blessing, giving rest and refreshment to the overworked. While we are young and healthy we can afford to be amiable; our troubles are few and light, and pass over us like water We think we shall never grow old; other people may, but they haven't our pluck. Our health and spirits will never be exhausted; we will take good care of that, and if not—well, "let us eat, drink, and be merry, for to-morrow we die." So thinks and speaks the soul untouched by the love of God, and goes on its way rejoicing. But as years pass on the tone begins to alter, for real troubles and cares accumulate; life is now a grind, a thing hardly worth having. The young man whose earnings have been more than sufficient for his wants and pleasures, becomes ill-tempered and irritable when he finds it hard to make both ends meet now there is a wife and family to support; or the wife on her side gets fretful, and he tires of meeting grumbling tones and crying children when he comes home from work, and the angry word and blow are the result. Then, poor soul, to mend matters, he tries to find amusement in the public-house! We all know the old proverb about the first step—the downward path is only too easy to tread, and we have often seen that the end of these things is death—death both of body and soul! Perhaps before this end is reached, the man has an accident while working, and is taken to the nearest hospital. Here, when he comes to himself, his thoughts turn to home, and he wonders how they will get on there, and if they are sorry for his misfortune, or glad to be rid of him. Then comes the after-thought. He is not sure he has been all he ought to have been to his family. His wife's visit, with her tear-stained face and unfeigned grief, and loving messages from the little ones, awaken all his better self, and he resolves, please God, if he gets over this, to be a kinder father and husband in the future. These good resolutions are fostered by the sympathy and care of all about him, and when the first agony of pain is passed and recovery sets in he enjoys the unaccustomed luxury of reading, or, if he is not much of a reader, comfortable chats with the other patients make the time go quickly by

These first steps towards a better life are something, but not much unless backed up by true repentance, and humble and hearty prayer to the Great Father to help him keep his good intentions, and turn back from the broad way which leadeth to destruction.

We will take another case of a poor overtasked wife. Often weak and sickly, with many a mouth to feed, husband out of work, and the rent behindhand, is it wonderful she should lose heart and perhaps let some duties slide which she might keep up? She toils on, striving against her weakness, until one day she breaks down altogether; then, though the neighbours are kind and do their best, it is no use. She must have care and attention, and food beyond what she can get at home, and so she, too, is taken to a hospital. Much against her will, be it said, for how can father fend without her, and Jane be sure to send the children to school with clean hands and faces; and little Tommy, who is addicted to "tantrums," will be a handful without mother to keep him straight.

Many a one has been stopped from evil courses by, what appears to them at first, an awful calamity. The vain girl is saved by a scarred and pitted face from going wrong; the silly youth, crippled by an accident, can no longer join in sports that were ruining him; the hardened sinner finds in paralysis space for repentance. Let us who have health and strength take heed to our ways, and join in earnest prayer for all those "who are any ways afflicted or distressed in mind, body, or estate." It takes a long time to reconcile us to God's will. The knowledge that we are not left to ourselves, but have a loving Father who gives His angels charge concerning us, and fellow-creatures ready with sympathy to lighten our loads, will help us forget our worldly griefs, and lead us to set our hope on things above, where true joys only are to be found.

